# Surgical treatment and outcome of pulmonary hamartoma: a retrospective study of 20-year experience

**DOI:** 10.1186/1756-9966-27-8

**Published:** 2008-05-31

**Authors:** Wei Guo, Yun-Ping Zhao, Yao-Guang Jiang, Ru-Wen Wang, Zheng Ma

**Affiliations:** 1 Department of Thoracic Surgery, Institute of Surgery Research, Daping Hospital, Third Military Medical University, Chongqing 400042, PR China

## Abstract

**Background:**

Controversy still exists in the indication and timing of surgical treatment of pulmonary hamartoma (PH). The objective of this study is to summarize the experience and the outcome of the surgical treatment for pulmonary hamartomas, and to assess the effectiveness and necessity of surgical therapy administered in patients with pulmonary hamatoma as well as clinical and pathological features and long-term follow-up results.

**Methods:**

This retrospective report has reviewed a 20-years clinical history of surgical treatment for 39 patients with PH from 1985 to 2006. These thirty-nine patients underwent 40 operations as follows: wedge resection (23), enucleation (10), segmentectomy (3), lobectomy (3), and pneumonectomy (1).

**Results:**

The PH occurred most frequently (78.1%) in the patients aged 40 to 60 years and the sex ratio (male/female) was 2.25/1 in our series. No postoperative death was encountered. One patient with pleural effusion was cured after thoracentesis. All of these 39 patients were proved with pathologic diagnosis of PH and the popcorn calcification was found in 6 cases before operation. In 38 cases having the mean follow-up of 7.3 years, a patient was operated thrice for regional recurrence.

**Conclusion:**

Fast frozen section in operation is critical for acquire accurate pathological diagnosis. Due to potential trend of recurrence or malignance, patients with hamartoma should be submitted to a complete evaluation and a regular follow-up.

## Background

The incidence of pulmonary hamartoma (PH) in the general population was 0.25% [1]. In the patients with benign tumor of the lung, hamartoma was the most common and accounting for 3% of all tumors of lung [2], and 6% of solitary pulmonary nodules [3]. According to the autopsy results from the Mayo Clinic, PHs were found in 2 of 7, 972 cases (0.025%) [4]. Another large autopsy series study from South Africa displayed that PHs were found in 152 of 47, 635 male coal miners (0.32%) [5]. PH was usually composed of a mixture of cartilage, fat, fibrous tissue, and smooth muscle and of branching clefts of nonneoplastic reactive bronchial-type epithelium. Actually, the word "hamartoma" deriving from Greek terms meaning "error" and "tumor" [6], was first used by Albrecht [7] in 1904 to describe lesions that contained an abnormal mixture of normal components of an organ [6-8]. PH was thought to arise from embryologic rests that were present in fetal life but generally did not become visible until adulthood. But a cytogenetic analysis of the PHs showed an abnormal karyotype and revealed recombinations between chromosomal bands 6p21 and 14q24 [9], thus supporting the opinion that a hamartoma of the lung was a true neoplasm. However, the nosogenesis of PH remains unclear. Most clinical studies about PH focused on the difference to malignant lesions and the diagnosis measures with effectiveness and specificity. Today, even with the advancement in medical therapy, pulmonary resection remains the most important measure of treatment of patient with hamartoma of lung. Controversy, however, still exists about the indication and timing of surgery [10-12], and the incidence of malignance or recurrence occurred postoperatively is still under question [13,14]. Herein we analyzed a 20-year historical series of patients with PHs who underwent surgical resection in our hospital, and assessed the effectiveness and necessity of surgical therapy administered in patients with PH as well as clinical and pathological features and long-term follow-up results.

## Methods

From January 1985 to December 2006, 39 consecutive patients with PH underwent surgical resection in the Department of Thoracic Surgery, Daping Hospital. The medical records of these patients were reviewed before surgery. The information about the age and gender of patients, course of disease, initial manifestations or symptoms, history of tobacco consumption, calcification in the hamartoma, location and size of the lesions, operative procedures, surgical complication, hospital stay after tumor resection and outcome was recorded. Intraoperative frozen section examination was performed in 32 of these patients (82.1%) and the histological diagnosis was obtained postoperatively from the resected specimen. To reconfirm the diagnosis of PH, the same pathologist reviewed all available histological slides. All patients had clinical follow-up by the same team of surgeons. Stable clinical outcome was also confirmed by telephoning the patients. The mean follow-up was 7.3 years (range: 1.5–14 years). Numerical data were presented as the mean ± SD. The difference between means was performed with ANOVA. All statistical analyses were performed using SPSS11.0 software (Chicago, IL, USA) and *p *< 0.05 was considered as statistically significant

## Results

In our series, 27 (69.2%) patients were male and the sex ratio (male/female) was 2.25/1. There was significant difference in sex ratio between male and female patients (*P *< 0.05). The mean age at surgery was 48.9 ± 13 years (range: 25–69 years). Twenty-eight of the patients (71.8%) were between 40~60 years and only one patient (3.2%) was under 30 (25 years old). Significant difference was also observed in different age group (*P *< 0.01) (shown in Table 1). And PH occurred most frequently (71.8%) in the patients aged from 40 to 60 years in our series. The course of disease ranged from 0.5 month to 6 years (mean of 1.56 years). Thirty (76.9%) of our patients had chest symptoms such as coughing, expectoration and thoracalgia. In these 30 patients, hemoptysis was encountered in 4 cases (10.1%), pyrexia in 4 (10.1%), and hypodynamia in 3 cases (7.7%). The remaining 9 patients (23.1%) were asymptomatic at the time of diagnosis (shown in Table 1). No respiratory infection or obstructive pneumonia due to obstruction of the bronchus was encountered in our series. One patient had accepted gastric myomatectomy before admission, one had the history of thoracic injury, and one had multiple renal cysts. Chest X-rays examination was performed preoperatively in all cases, the most common manifestation of chest roentgenogram was an irregular high-density margin with small lobulations located peripherally. In terms of the location of lesion, there was no significant difference between the right and left lung (*P *> 0.05). The mean diameter of tumors was 2.9 ± 1.6 cm (range: 1.5 – 8.5 cm). Most of the PHs in our series (53.8%) had the diameter ranged from 2.0 to 3.0 cm, 13 of them (33.4%) were under 2.0 cm and only 5 (12.8%) over 3.0 cm (shown in Table 2). Calcification formation was found in 15 cases (38.5%), only 6 of them (15.4%) were the typical popcorn calcification, and the remaining 9 (23.1%) had patching calcification on chest roentgenogram. Chest computed tomography performed by radiology department in our hospital was obtained in 17 patients, and the other 19 patients accepted CT examination at other hospitals. So there were 36 patients had CT scan. Due to financial reasons, the remaining 3 patients didn't accept this expensive examination.

In all of these patients in our series, there were 38 cases accepted 38 operations, including 23 wedge resections (4 cases had video-assisted thoracoscopic surgery), 10 tumor enucleations, 3 segmentectomies and 2 lobotomy. The remaining one patient accepted tumor enucleation in local hospital at age of 25 years, the tumor located in upper lobe of right lung with pathological diagnosis of chondromatous hamartoma. Regional recurrence was found and resection of tumor was performed twice in 9 years after the first operation. The patient underwent tumor enucleation and right upper lobectomy respectively. However, similar lesion occurred again at the ipsolateral lung after the third surgical treatment. Because of three enormous tumors (with diameter over 8.0 cm) occupying the remaining middle and lower lobe of right lung, a pneumonectomy had to be performed. Moreover, because of severe adherence and disturbances of blood coagulation, the intraoperative hemorrhage was hard to control. The total amount of blood transfusion was 14, 700 ml (including 3, 900 ml banked blood and 10, 800 ml of reclaimed blood). Fortunately, this patient recovered smoothly after operation without any complication. No patient died in the postoperative period or during the follow-up period. Postoperative pulmonary atelectasis occurred in one patient at the operation day, and the blood coagulum obstructing the right principal bronchus was found by emergent bronchofibroscope examination. After removing the clot, the breath sound of right lung recovered. Due to inadequate drainage, pleural effussion was encountered in one patient and was cured by thoracentesis. Follow-up continued until March 2007, ensuring a minimal follow-up of one and a half years. Thirty-eight patients submitted the regular follow-up, but one patient lost touch after discharging. The mean follow-up was 7.3 years (range: 1.5–14 years). Recurrent disease developed three times in one patient (2.6%) after enucleating of the tumor. At the end of follow-up, none of the 38 patients died, and the concomitant malignant lesion was not found in our series after operation.

## Discussion

PH occurs most frequently in the patients aged from 40 to 70 years, with a variation in male preponderance from 2: 1 to 4: 1 [4,15], this maybe due to the higher morbility of pulmonary disease in male. In our series, the ratio was 2.25: 1, and 21 patients (53.8%) had heavy smoking history. Twenty-eight of our patients (71.8%) were between 40~60 years and only one patient was under 30 (25 years old), which was consistent with other studies. PH could occur in all parts of lung, but most often in the periphery and rarely near the hilar parts [16,17]. Endobronchial hamartoma was reported in approximately 3% to 19.5% cases [10,17-19], but not been found in our series. Hamartomas are usually solitary, well-demarcated nodules on roentgenogram. Multiple hamartoma are a rare entity and only 19 cases have been described in the world [20-22]. Slow growth is the norm for PH, malignancy is rare and only rare cases have been reported [23,24]. PHs are predominantly composed of cartilage; other components include fibromyxoid connective tissue, fat, bone and smooth muscle [25]. Usually, the PH has non-specific clinical manifestations and was detected by chest radiographic examination occasionally [4,11,16,25]. The respiratory infection or pulmonary atelectasis due to mechanical obstruction of the bronchus could be encountered in part of patients with PH. In our series, part of patients (23.1%) were asymptomatic at the time of diagnosis, and which supported the concept that clinical manifestation could not be the effective method or standard of the diagnosis for PH.

Although hamartoma was generally considered to be a benign neoplasm, there have been several reports of increased risk to lung cancer in patients with a chondromatous hamartoma and the relationship between hamartoma and the synchronous malignancy was still unclear [23,26,27]. Tomiyasu *et al *[28] reported a unique hamartoma that penetrated the visceral pleura, and after surgical resection, a new bronchial carcinoma had arisen at the same site. Kato and his colleagues found a postoperative recurrence in a patient with multiple pulmonary leiomymatous hamartoma [29]. Moreover, similar recurrence of PH was also encountered in our study. A male case underwent tumor resection three times for regional recurrence. Eventually, right pneumonectomy had to be performed due to three recurrent enormous tumors. The results in our series also manifested that the rarity of PHs really had the tendency of malignance.

**Figure 1:**
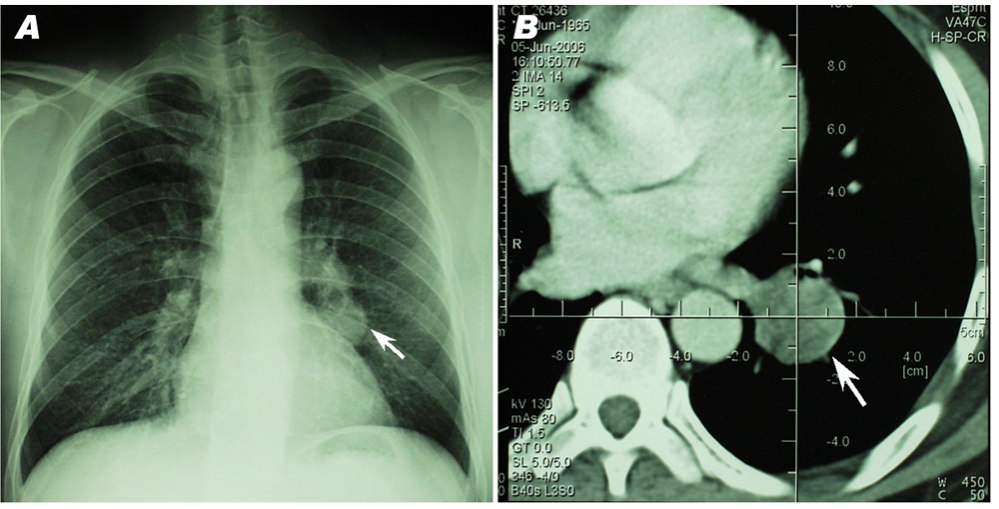
**Imageological examination of one patient**. Chest X-ray (A) and CT Examination (B) showed a solitary nodule over left lower lobe, with irregular margin and small lobulations, which could not be differentiated from lung cancer before operation.

Because the symptoms of patients with PH were frequently indistinguishable from those of a carcinoma, radiological examination was necessary for diagnosis of PH. The PHs tended to be small (less than 4 cm), round, peripheral, well-circumscribed tumors that occasionally showed "popcorn" calcifications on chest radiograph. They were usually asymptomatic and discovered incidentally in radiologic studies [30]. All of the hamartomas in our series were representing as intrapulmonary mass shadow without pleural indentation sign, sublobe or sentus. Calcifications were found in 15 cases and only 6 of them were the typical popcorn calcification. As the characteristic appearance on chest radiograph, popcorn calcification was important for diagnosis of PH. However, it could not be the definitive diagnostic evidence since calcifications may appear in carcinomas and in tuberculosis as well. It was also observed that the lesions of some cases displayed with solitary nodules, which could not be differentiated from lung cancer before operation (shown in figure 1).

Aspirates of PH were often scanty because of the dense nature of the lesion. And the diagnostic accuracy has been shown to be less for PH than for malignant tumors, partly because of the difficulty in obtaining diagnostic material from cartilaginous lesions and partly because of difficulty in the interpretation of the findings [30-33]. Considering the risk of pneumothorax and the potential delaying of possible malignant lesion, and because all patients in our series had the indications of surgery, aspirates was not performed in our study.

Today, even with the advancement in medical therapy, pulmonary resection remains the most important treat measure of patients with PH. Controversy, however, still exists about the indication and timing of surgery [10-12]. Since most PHs are nonexpanding or slowly growing neoplasms, some authors believed that surgery was necessary only when expansion was recorded in young or middle-aged patients or accompanying obvious pulmonary symptoms. In our opinion, the PH did have the tendency of expansion or recurrence, and chronic inflammatory stimulation of local position might contribute to the development of malignancy. Therefore, when a solitary pulmonary lesion was more than 2.5 cm or the possibility of malignancy could not be excluded, surgical resection was deserved to be performed and should be mandatory. In terms of indications of surgery, the author presumed to conclude as follows: (1) a solitary pulmonary lesion with diameter more than 2.5 cm; (2) overweight psychic burden making it necessary to remove the lesion; (3) having the tendency of expansion or recurrence; (4) with pulmonary symptoms unresponsive to drug treatment; (5) above all, the lesion could not be differentiated from malignancy. Especially to the patients in the fifth section, any unwiring delay would minimize their chances of acquiring long-term survival.

## Conclusion

Wedge resection was the main choice in our series for patients with PH. More aggressive lobectomy or total pneumonectomy was considered only in such status: (1) the central intrapulmonary hamartoma located in the deep part of pulmonary lobes and adhered severely to hilum of the lung, (2) the distal lung tissue was nonfunctional, (3) multiple or huge tumors making wedge resection impossible. To avoid ignoring potential malignant lesions, the necessity of the intraoperative frozen section must be emphasized. Regarding the principles of the surgery, we believed that normal lung tissue should be reserved as much as possible. Furthermore, due to lack of the malignance after operation in our series, the author presumed that the wedge resection was safe enough. However, due to the potential trend of recurrence, it must be emphasized that the patients with PH should be submitted to a complete evaluation and regular follow-up.

## Competing interests

The authors declare that they have no competing interests.

## Authors' contributions

WG collected the data and drafted the manuscript, ZY carried out the statistical analysis, JY designed this study and modified the manuscript, WR participated in its design and coordination, MZ helped to carried out the statistical analysis. All authors participated in performing the operations and approved the final manuscript.
